# Retraction Note: Preliminary risk assessment of in-vessel leakage accident in ITER

**DOI:** 10.1038/s41598-024-65933-1

**Published:** 2024-07-01

**Authors:** Qizhi Duan, Xingchen Fang, Shuai Chen, Hongyun Xie, Chunbing Wang, Dagui Wang

**Affiliations:** 1 State Key Laboratory of Nuclear Power Safety Monitoring Technology and Equipment, China Nuclear Power Engineering Co., Ltd., Shenzhen, 518172 China; 2 Anhui Jianzhu University, Hefei, 230601 Anhui China; 3 Hefei Institutes of Physical Science, Chinese Academy of Sciences, Hefei, 230031 Anhui China

Retraction of: *Scientific Reports* 10.1038/s41598-024-53304-9, published online 12 February 2024

The Editors have retracted this Article.

After the publication of the Article concerns have been raised regarding the usage of internal ITER data without proper permissions. Additionally, it was brought to Editors attention that the data used was outdated and that there is a lack of supporting evidence for evaluations such as dose calculations and the causes and frequency of the in vessel events that led to incorrect conclusions. The Editors therefore have lost confidence in the results and conclusions presented in this Article.

All authors agree with the retraction and its wording.

